# The diagnosis of primary hyperparathyroidism in developing countries remains in the past century: still with bones, stone and groans

**DOI:** 10.20945/2359-3997000000240

**Published:** 2020-04-27

**Authors:** Marise Lazaretti-Castro

**Affiliations:** 1 Divisão de Endocrinologia Escola Paulista de Medicina Universidade Federal de São Paulo São Paulo SP Brasil Divisão de Endocrinologia , Escola Paulista de Medicina , Universidade Federal de São Paulo , São Paulo , SP , Brasil

The existence of primary hyperparathyroidism (PHP) as a disease was first recognized in the beginning of the last century. After J. B. Collip had discovered the physiological role of parathyroid glands on calcium homeostasis (1), other researchers identified increased parathyroid glands in patients with *osteitis fibrosa cystica* and osteomalacea (2). Then, the first parathyroidectomies began to be performed in order to treat PHP in patients severely affected by the disease (3). From that time until now, there has been a great gathering of knowledge about this disease, which remains challenging us in its clinical presentation, diagnosis and treatment.

PHP in its classical form is characterized by hypercalcemia with inappropriate parathormone (PTH) concentrations, which is not suppressed, as expected, in the presence of high calcium levels (4). It is usually caused by a single parathyroid adenoma, but also can be due to a hyperplasia of multiple glands and, more rarely, adenocarcinomas. The classical picture present bone disease, with pain, brown tumors, deformities and fractures ( *osteitis fibrosa cystica* ), nephrolithiasis, nephrocalcinosis and manifestations of hypercalcemia, such as nauseas, vomiting, weight loss, polyuria and polydipsia, dehydration, renal insufficiency and neurological symptoms as depression and cognitive impairment (5). Because of this diverse and severe clinical picture, it was defined in the past as the disease of “bones, stones and groans”.

Nevertheless, in the last 50 years its clinical presentation has been changing, and less symptomatic forms were recognized as much more frequent than previously thought (5). As laboratory and imaging tests become more available to the general population, the diagnosis becomes more sensitive. Currently, in well developed countries, PHP can be considered the 3 ^rd^ most common endocrine condition, just after diabetes mellitus and thyroid diseases (6). The great increase in the incidence of PHP comes along with the implementation of routine biochemical screening, which included calcium determination (5). In addition, an improvement in PTH assays, with the development of immunometric methods to measure the intact molecule, has increased accuracy and facilitated the diagnosis (7). Therefore, in USA and Europe the so called asymptomatic PHP is the most common presentation of this disease.

Nevertheless, in developing countries, the reality seems to be different and symptomatic forms are still more frequent, although changes on their clinical presentations are being describing by some. Yadav and cols. (8) bring this discrepancy to discussion in a systematic review in this issue of AE&M. The authors’ first finding was the small number of publications on the epidemiology of PHP in developing countries, compared to the large volume of publications on the same topic found in developed countries, whose greatest concern now is the approach to the asymptomatic forms. The authors pooled 17 studies from 11 countries, nine of them are from Asia and only 2 from west side (Brazil and South Africa). Brazil emerged as the country with the highest prevalence of asymptomatic cases, but still about half of the patients presenting with symptoms. In the other countries, the percentage of symptomatic patients is quite high, reaching 100% in studies in India and Iran, with a mean of 80% with symptoms from the total population of the study. The most symptomatic patients are younger and present higher mean levels of PTH and total calcium, compared to developed countries. In 5 studies it was possible to follow the increment in the number of diagnostics in the last years, with patients becoming less symptomatic and older over the years of observation.

Vitamin D deficiency has been proposed as one possible cause for more severe PHP presentations in developing countries (9). The present review confirms that this deficiency is a common condition, since the mean levels of 25 hydroxyvitamin D (25OHD) was 20.0 ± 22.6 ng/mL. In PHP, lower levels of 25OHD induce additional increments on PTH and bone remodeling markers and the replacement of Vitamin D is followed by a decrease on these biomarkers, which could improve bone quality (10,11). However, the consequences of the Vitamin D replenishment on the severity of bone disease still need to be confirmed and seems to be unlike to justify just the discrepancies on clinical presentations as compared with developed countries (11).

The changes in the profile demonstrated by Yadav and cols. over time in some countries, with an increase in less symptomatic forms, whereas they became older and with mild biochemical alterations, sounds much more in favor of a lack of diagnosis than a different form of the disease in these sites. The symptomatic form being more frequent in younger could be linked to some genetic disorder, and it is possible that these severe forms will always exist as a rare disease. An increment on milder forms of PHP in developing countries, as was witnessed in USA and Europe, will only be possible when a routine screening for blood calcium is implemented. Otherwise, an asymptomatic disease will never be discovered, if not investigated.

It is well known that these “asymptomatic” forms may already show some renal changes, such as nephrocalcinosis, when carefully investigated, besides an increased risk of cardiovascular disease in the long term (4), therefore it is essential improving the diagnosis. To do so, it is important to advise general practitioners about the importance of this diagnosis and to have a well-trained team of endocrinologists and neck surgeons in order to proper treat this condition. In addition, it is mandatory to include blood calcium measurements as a routine procedure, especially in the elderly, the population at greatest risk for the milder forms of primary hyperparathyroidism. And, at last but not least, this review showed us the urgent need of higher quality epidemiological data from developing countries to identify possible regional characteristics and to improve health policy strategies for PHP diagnosis and treatment. Only then will developing countries be able to emerge from the era of bone, stone and groans.
